# Contextual adaptation, implementation, and outcomes of individual placement and support: a case study

**DOI:** 10.1186/s43058-026-00875-5

**Published:** 2026-02-05

**Authors:** Suzanne Johanson Sturesson, Ulrika Bejerholm, Marcus Knutagård, Verner Denvall, Kristina Carlsson Stylianides

**Affiliations:** 1 Department of Neurosciences and Physiology, The Sahlgrenska Academy, Gothenburg University, Gothenburg, Sweden; 2 Department of Health Sciences, Medical Faculty, Lund University, Lund, Sweden; 3 Department of Urban Studies, Malmö University, Malmö, Sweden; 4 School of Social Work, Faculty of Social Sciences, Lund University, Lund, Sweden

**Keywords:** Adaptation, Implementation science, Core functions, Mental health service, Evidence-based practice, Individual placement and support

## Abstract

**Background:**

Implementing the evidence-based Individual Placement and Support (IPS) model in welfare states like Sweden faces contextual challenges that often necessitate local adaptations to enhance fit and effectiveness. While fidelity assessments aim to ensure effective outcomes, adaptations may lead to unintended drift from core components. The Model for Adaptation Design and Impact (MADI) help analyse the characteristics of adaptations and their ripple effects. This study explores the interplay between implementation, adaptations and fidelity in mental health services using the MADI framework.

**Methods:**

An embedded case study design was used, involving five mental health services in a major Swedish city. Data from 26 key informants, field notes, and meeting protocols were analysed using content analysis and interpreted through the MADI framework.

**Results:**

The adaptations were both planned and unplanned, some adaptations supported the integration of IPS and others negatively impacted core components, revealing unintended impacts on implementation and intervention outcomes.

**Conclusions:**

To understand adaptations and its pathways when implementing IPS in mental health services, and other stakeholders, the MADI framework is valuable. Excessive adaptations can compromise core values and fidelity assessments must account for adaptations and their consequences. To ensure IPS remains effective, some practices within mental health services and other organisations may need to be revised or adapt to the method itself to support IPS implementation and outcomes. The findings offer valuable insights for professionals and user organisations adopting evidence-based practices in mental health services.

**Supplementary Information:**

The online version contains supplementary material available at 10.1186/s43058-026-00875-5.

Contributions to the literature

- The use of the MADI framework for adaptations during implementation provides a robust explanatory model for how interventions are adjusted to fit the local context. This is particularly relevant for IPS in mental health services that, despite strong evidence, has proven difficult to implement.
- The study offers insights into the consequences of extensive adaptations, highlighting the risk that such modifications may compromise the core components of an intervention.
- Considering whether de-implementation of existing methods should occur in parallel with the implementation of new ones is essential, thereby offering an incentive to map and reflect during the process.

## Background

Adaptation is considered necessary during implementation [1, 2], however, applying interventions that contrast the implementation context can prove to be particularly challenging, as core values related to evidence might be affected [3]. Research evidence for the effectiveness of Individual Placement and Support (IPS), for people with severe mental illness seeking competitive employment is explicit, with an employment rate around 55% among 28 randomised controlled trials [4–8]. In Europe, implementation efforts are currently being shared and developed through the IPS European Learning Community, an international network of members who, in various ways, make policy, work with or conduct research on IPS [9]. while maintaining a close connection with the American founders of IPS. The association has compiled research and implementation experience from multiple countries, showing that IPS implementation across Europe remains uneven due to differences in welfare systems, government services, cultural contexts, and persistent barriers. IPS constitutes a complex intervention, according to Medical Research Council guidance [10, 11] as it involves individual support, collaboration with psychiatric teams, and coordination with external stakeholders such as employment services, insurance authorities, and employers. Consequently, implementation barriers can emerge at multiple levels and in diverse settings. Continued examination of these contextual barriers is therefore essential to promote sustainable implementation within complex interventions and welfare systems [3, 12–18]. Although many barriers have been identified in current research, adaptations have received less attention, especially concerning unplanned adaptations and consequences of such [2]. Understanding the effects that barriers and adaptations may lead to would make it easier for researchers and practitioners to anticipate and address those adaptations that do not actually benefit the intervention itself.

It has been generally maintained that adaptations are necessary during implementation, as described in several guidelines and frameworks [1, 19–21]. Deliberate and well-planned adaptations may lead to a better fit and effectiveness in a local context [22]. Adaptation as a concept, includes planned adaptations, stringently analysed in relation to perceived contextual barriers and unplanned adaptations, taken as decisions by co-ordinators or managers [19]. Negotiations which can occur daily in the realisation of certain methods, are also common. Even though adaptations are necessary, both planned and non-planned adaptations might have unintended consequences on the intervention and implementation outcomes, which has been well described and developed in the Model for Adaptation Design and Impact (MADI) [2]. Some adaptations counteract the core of the intervention while others only have a marginal effect [1]. Adaptations and fidelity have been proposed to co-exist [1, 23], where fidelity assessments track intervention adherence and prevent deviations from core functions [1, 24]. Organizational demands may dominate when fidelity is low thus impacting intervention delivery [18]. This could be the case in a single organisation but also when an intervention overlaps several different organisations, which is common in welfare structures.

Previous research show that it is essential to consider structures that support organizational change when disseminating the IPS model to different welfare contexts [25]. Furthermore, attitudes toward individuals with mental illness and their opportunity to achieve employment need to be addressed, together with the importance of adapting employment regulations and insurance benefits [25]. Inherent contradictions exist in the Swedish welfare context between the commonly practised stepwise vocational rehabilitation model and the principles of IPS, as well as the recovery perspective [12, 13]. Stepwise vocational rehabilitation entails pre-employment assessments of functioning and work ability, and individuals may be placed in various activities to practice skills. In contrast, IPS emphasises rapid entry into competitive employment and provides training directly in the participant’s chosen workplace (see Table 1). Stepwise rehabilitation is often prolonged due to these assessments and because individuals are referred to internships with fixed, and often lengthy, durations set by authorities.

**Table 1:** IPS principles guiding the intervention

IPS principles
Everyone who wants to work is eligible for IPS participation
Job seeking starts early in the process
Follow service users´ preferences
Achievement of competitive employment
Personal information about insurance benefit
Integration of IPS in the mental health service team
Build a network of employers
Tailored support at work

Core IPS principles, including early job search initiation, adherence to service users´ preferences, eligibility determination, and achieving competitive employment were identified at an early stage as being at-risk during implementation within the Swedish welfare system [12]. If traces of the stepwise model persist during IPS implementation without a modification of organizational structures, they may undermine core functions. Notably, some organizations maintain entire systems with formal routines that discourage IPS [15]. However, successful implementation has been achieved in mental health and community services, with good to high fidelity, despite these challenges [3, 12]. The reported implementation difficulties have primarily been related to organizational fit, authority regulations within vocational rehabilitation, and the need for system leaders to collaborate from the outset [13, 15]. Integration of welfare authorities beyond mental health services seems necessary, without adapting too much to external contexts. Intense collaboration among welfare authorities and employers, informed by a shared understanding of policy related to service users´ support needs, thus remains crucial [12, 16–18].

Implementing IPS in the Swedish welfare system remains challenging, not only because of contextual barriers but also due to adaptations to existing organizational practices that may undermine core components and reduce effectiveness [12, 15]. Greater understanding of how IPS is adapted within non-evidence-based vocational systems is needed to determine when adaptations begin to compromise the intervention’s evidence base and the support intended for clients. The overarching aim of the study was to explore adaptations of the Individual Placement and Support in five mental health services during implementation. The study strove to gain a better understanding of how adaptations might affect core functions of IPS. The following research questions were formulated: What characterizes the adaptations in the implementation process of IPS when applying the MADI framework? In which ways do the adaptations affect core functions, intervention and implementation outcomes?

## Methods

This study had an embedded case study design [26] and was part of a larger project where the implementation of the evidence-based practice Individual Placement and Support was followed. In this study five different mental health services were included and studied. A case study design was chosen to be able to follow the implementation process closely over time and to identify which type of adaptations took place. The embedded case study design was deemed appropriate as the implementation of IPS was a single project, including five different mental health services independently organized, which subsequently constituted several units of analysis [26]. Adaptations of the IPS model were studied both from a project perspective and by analysing data from the five mental health services, and in addition analysed retrospectively by applying a theoretical framework. To affirm the quality of this study, in terms of collecting, analysing and reporting the data, the COREQ checklist was used [27].

### The case

The “Integration of IPS in Psychiatric Care” program ran from 2019 to 2022 and involved collaboration between multiple organizations, including the Financial Coordination Association, local labour market administration, five different mental health services geographically situated in different parts of a greater city, the Swedish Public Employment Service (SPES), and the Swedish Social Insurance Agency (SSIA). The goal was to implement IPS for people with psychosis, focusing on employment support. Led by a project manager from the municipal labour market administration, the project included a steering group with representatives from all partner organizations and user groups. An implementation group was formed early to ensure sustainability. Financial agreements between the municipality and mental health services were pre-established by local politicians with the intention to sustain the IPS intervention. Each mental health service had a full-time IPS employment specialist (ES), trained and experienced in IPS, with a caseload of 20 clients. One mental health service had two ESs sharing a full-time role. The implementation followed a consistent process across the five mental health services, with regular meetings involving the project leader, mental health service managers, ESs, and treatment providers, along with IPS training at each mental health service.

### Conceptual framework

The MADI framework [2] has been developed within implementation science to better understand adaptations and intended as well as unintended effects (i.e. ripple effects) of adaptations in implementation processes. It is built on previous frameworks that have captured implementation characteristics [19], implementation outcomes [28], and possible causal pathways [19, 29]. Unlike many earlier implementation frameworks, MADI goes beyond description by helping practitioners and researchers examine relationships between adaptations and their outcomes, including potential mediators and moderators [2]. Mediating adaptations do not alter the core intervention, while moderating adaptations may affect its integrity and the evidence put to practice may be challenged. MADI was chosen for its ability to explore how core IPS principles are influenced by contextual adaptations, offering both nuance and structure to the analysis. As the framework is still evolving further research is needed to validate its use [2].

### Selection of case and informants

Informants were recruited purposely based on their role in the implementation process in the various organisations with the intention to capture diversity of experiences and perspectives of adaptations and the development of the implementation process, as well as to ascertain richness of information [26]. Five mental health services were in focus in this case, which covered various numbers of teams and different kinds of professional competencies and years of experience of working with mental health service, the mental health area or vocational rehabilitation. Employment specialists, the project leader, the head of the financial coordination association, mental health service managers, managers from the local labour market administration, key staff at the services such as occupational therapists, rehabilitation coordinators, social workers and case managers, were thus selected. The representative from the social insurance agency the public employment service was not interviewed because their representatives were replaced several times during the project, and they did not always participate in steering group meetings as they were undergoing a major reorganization during the project period. All participants received an email in which they were asked whether they would consider taking part in the study and being interviewed. None disagreed. The participants who were interviewed were provided with written and verbal information of the study, how data would be stored and that both the mental healthcare service and participants would be anonymised. They were also informed that this study was part of a larger interdisciplinary study, the reasons for conducting the study and the content of the interview guide. The results of the study have been presented in an IPS conference afterwards. Each informant signed a written consent. The study was part of a larger project that was approved by the Swedish Ethical Review Authority (Dnr 2019–03232).

### Data collection

To ensure data triangulation, this study used multiple sources: individual and group interviews, memos from steering group meetings, fidelity assessments, and project documents. Semi-structured interviews were conducted with 16 key informants between 2019 and 2021, along with follow-ups involving the project leader and two groups of employment specialists (*n* = 10). Interviews, guided by the Theoretical Domains Framework [30] explored organisational, group, and individual perspectives on IPS implementation, collaboration with authorities, and influencing organisational factors leading to adaptations. All interviews were conducted by one of the authors, recorded, transcribed professionally, and lasted 30–90 min. Memos, meeting protocols and workflow documents were used as material collected during steering group meetings that the first author attended.

Fidelity assessments were conducted using the Supported Employment Fidelity Scale (SEFS) (SEFS) [31] at five points between 2019 and 2021. The project leader performed the assessments, validated in consensus meetings with experienced IPS researchers. The SEFS, based on eight IPS principles, see Table 1, includes 25 items rated on a 5-point scale (total score: 25–125). The SEFS scale covers staff, organisation, and service areas, categorizing fidelity from “not supported employment” (less than 73 points), “fair fidelity” (74–99 points), “good fidelity” (100–114 points), to “exemplary fidelity” (115–125 points) [31]. These assessments helped evaluate adherence to IPS principles and identify adaptations during implementation.

### Data analyses

The data were analysed stepwise using a content analysis with a deductive approach [32]. Initially, the research team identified overarching patterns to understand the implementation process, including barriers, enablers, and adaptations. Materials were organized chronologically to support case reporting. In an iterative process through repeated review of transcripts and audio files, relevant passages, i.e. meaning units, adhering to adaptation characteristics were highlighted [2]. The meaning units were then condensed and coded by the first and the last authors, manually [32] and were cross-checked with memos and project documents for consistency. The codes were grouped into subcategories, labelled according to content that had emerged. The subcategories were then placed under the category “Adaptations characteristics”. In the next step of the analysis, further identification of the domains from the MADI framework continued, trying to recognize possible mediators; if the adaptation aligned with core functions, and moderators, trying to explain why adaptations were made. The last step focused on the adaptations’ potential impact on intervention and implementation outcomes and whether this impact was intended or unintended. Fidelity assessments were analysed in relation to IPS principles, also to determine whether adaptations aligned with core functions, based on fidelity scores.

## Results

The findings are structured around project conditions, sub-categories of adaptation characteristics, and their effects on core IPS functions, intervention, and implementation outcomes. Moderators and mediators are also described. Three main adaptation characteristics were identified: Adaptations to SPES’ rules and resources, Adaptations to a stepwise social insurance structure and, Adaptations to mental health services’ day-to-day workflows and case management (Table 2).

**Table 2:** Selected fidelity ratings from year 1 and year 3

**Fidelity summary**	**Total score, year 1**	**Total score, year 3**
	*92p: aligns partly with IPS*	*103p: aligns well with IPS*
**Fidelity areas**	**Score at project start**	**Score at project ending**
Integration of IPS in mental health service through team assignment	4	4–5
Collaboration between ESs and key handling officers in SPES	3	2
Zero exclusion criteria	4	5
The mental health services focus on competitive employment	2	3
Rapid job search for competitive employment	4	4
Individualised job search	5	5
Job development – frequent employer contact	1	3
Competitive employment	1	2
Community-based services	1	1

Comment to Table 2: IPS principles adhering to zero exclusion criteria, rapid job search for competitive employment and individualised job search were assessed as high fidelity, concurrently it was shown that adaptations to context unintendedly impacted on service and client outcomes

### Adaptations to SPES’ rules and resources

On the steering group level, inter-organizational collaboration with SPES was problematic due to inconsistent participation from their representatives and internal reorganisation. This hindered decision-making about collaboration routines and having a flexible approach to SPES rules. IPS delivery was adapted to fit SPES regulations from start of the project. The ESs modified the IPS methods based on previous experiences in which the employment service’s legally mandated resources proved important for being able to support service users. These adaptations were thus unplanned, without decisions from the steering group, however, made with good intention. The resources available of the SPES authority (subsidised employment, educations, internships, employer network) were described as important as these enabled the support for the individual IPS service user to gain employment. ESs followed established routines, and thereby integrated SPES pathways into IPS, which was an adaptation to the stepwise approach. The tradition of following authority rules for return to work was described as strong. However, these efforts of cooperation were hampered by delays and communication issues, creating a complex structure that limited ES’ autonomy. They felt dependent on SPES´s resources but at the same time they were frustrated that working routines were complicated, and it impacted on the service users’ processes. Despite detailed and legitimate employment plans, the ESs often found themselves positioned as a subcontractor to the SPES.

### Intended or unintended impact on intervention and implementation outcomes

**Fig. 1:**
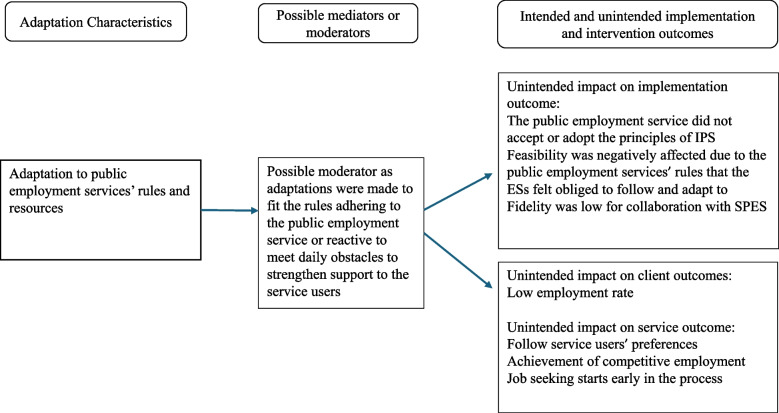
Adaptation characteristics and their impact on implementation and intervention outcomes in relation to public employment service

When ESs adapted to SPES’ rules, they often acted as moderators within existing systems to make IPS fit into the employment service. However, these adaptations deviated from IPS core functions of follow service users’ preferences, starting job search early in the process and achievement of competitive employment, see Fig. 1. Some adaptations were reactive, driven by daily obstacles, and were made to strengthen service users’ support. Collaboration with SPES remained weak, impacting unintendedly on implementation outcome with low fidelity ratings and inconsistent participation, which, in turn, impacted unintendedly on intervention outcomes related to job search efforts and shifts from competitive employment to internships. These adaptations, though well-intentioned, compromised the core function of service users’ preferences as they were referred to measures that were not in their interest and, delayed job search initiation and outcomes, because of long periods of waiting for internships or other measures.

A key strategy and to systematically enhance collaboration with SPES at the steering group level, failed due to poor engagement and reorganization at the SPES offices, affecting the implementation outcome feasibility and adoption and, in the end, client outcome. Some service users withdrew from the intervention without securing internship, employment or education. Of 153 participants, 27% gained employment, 12% pursued education, and 48% entered internships. However, employment rate was also affected by the Covid-19 pandemic, according to analyses by the steering group. Furthermore, the adaptation to SPES´s regulations had ripple effects. SPES-related adaptations increased administrative burdens for ESs, delaying personalized job matching.

### Adaptations to the stepwise social insurance structure

To secure economic benefits, ESs coordinated with SSIA for personalised information in line with core functions. Even so, adaptations to the stepwise approach were common as employment compensation implied that the IPS service user needed to undergo several assessment steps and participate in authority measures in line with the social insurance structure. These adaptations were unplanned and were not actively made by any actor; they were difficult to counteract because they are part of the authority’s regulations and the laws governing sick-leave processes.

However, the SSIA representative in the steering group made great efforts to smooth the pathway to employment trying to benefit the service users. Despite this, measures were added to the content of the IPS principles. ESs navigated complex regulations, often implicitly following authority regulations rather than IPS guidelines, such as waiting for assessments of individuals’ work ability.

### Intended or unintended impact on intervention and implementation outcomes

**Fig. 2:**
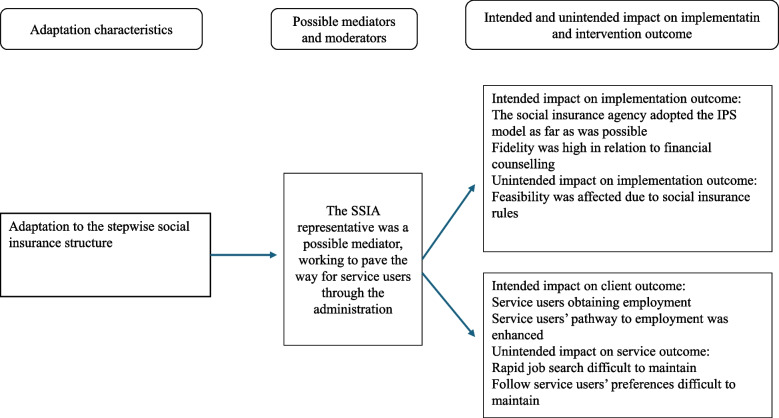
Adaptations characteristics and their impact on implementation and intervention outcomes related to social insurance structure

The SSIA involvement facilitated the implementation by adopting the model of IPS, especially during inter-organizational challenges. The representative from SSIA acted as a mediator, when trying to pave the way for the ESs to work in line with the core functions. Even though this was not always successful in certain individual situations it had an intended impact on intervention outcome in several cases. Extended timelines compromised core functions like rapid job search and individualised jobs. The adaptation to authority regulations was needed to fit into national laws but difficult for the project to manage as IPS principles follow a different order than the regular rules. Implementation outcomes were unintendedly affected by these adaptations, with an impact on feasibility. Fidelity was high throughout the project as service users were provided with financial counselling about forthcoming salary in relation to financial benefits. Despite strategic planning by the steering group and a SSIA representative acting as a champion and mediator, integrating IPS into SSIA proved difficult in practice, see Fig. 2.

### Adaptations to mental health services’ day-to-day workflows and case management

Adaptations varied across the five mental health units. While four units were ready for IPS implementation, existing routines largely remained unchanged. The ESs integrated into the services by adapting to existing workflows rather than transforming them. This was a slow process and an unplanned adaptation, as working in the mental health services’ organization was a new experience for the ESs. Staff-intensive collaboration was difficult due to entrenched practices, such as weekly team meetings without service user involvement. ESs participated in these meetings with the intention to collaborate around the service user but often had to seek out collaboration informally. They valued proximity to the team but were frustrated by limited opportunities for direct, collaborative work with service users as in line with IPS.

Instead of asking each service user whether they wanted to participate in IPS to receive support in gaining employment, the ESs initially encountered service users deemed “not ready” for IPS, by treatment staff, due to long-term sickness benefit or the mental health condition. Often these service users also perceived themselves not being able to work. Staff typically referred such service users to SPES, prompting ESs to assess whether IPS support was feasible. This led ESs to another unplanned adaptation, into a routine that was more medicalised and followed the stepwise approach, aligning with existing mental health practices. Both treatment staff and ESs advocated for the service users but hade different approaches. The adaptations to mental health service’s routines generally occurred in an organic way. Over time, the ESs attempted to introduce the practice of asking all service users whether they wanted support in gaining employment, but this never fully took hold. Likewise, during the project, it was not possible to change the routines of team meetings so that service users would participate in planning and discussions.

**Fig. 3:**
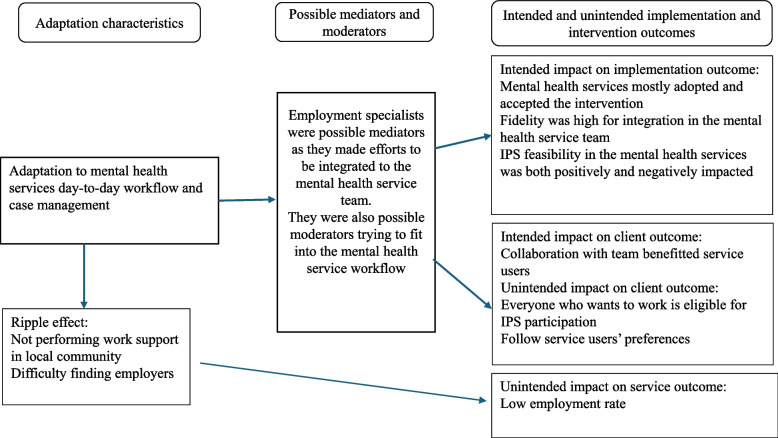
Adaptations characteristics and their impact on implementation and intervention outcomes related to the mental health services

At one unit, IPS integration was delayed by two years due to resistance from case managers. Several ESs resigned from the project due to exclusion. The situation prompted project leaders to convene and initiate changes and there was a gradual shift toward offering employment support to service users, though IPS remained heavily adapted to the case management model. There were limited user engagement and traditional roles like referrals and assessments persisted. For description of adaptation characteristics, possible mediators and moderators, as well as impact on implementation and intervention outcome, see Fig. 3.

### Intended or unintended impact on implementation and intervention outcome

The ESs’ efforts to collaborate as a team around the service users by seeking out staff outside the routine meetings served as a way of acting as mediators to uphold the core function of being integrated to the team. This had an intended impact on client outcome as it benefited the service users because the collaboration with the staff made the planning for work clearer and made the core function of integration into teams more obvious.

Managers at mental health services declared that they adopted IPS early on. Local project groups were established at each unit and internal goals were set. However, broader staff engagement was limited. IPS training was rather brief, leaving staff unclear on ESs´ role. Staff continued to select participants for IPS, and ESs acted as possible moderators as they made efforts to fit into the workflow and tried to bypass collaboration hindrances. This adaptation contradicted the core function of zero-exclusion, and it had an unintended impact on the intervention outcomes; the number of service users being included in IPS and thereby, the number of service users obtaining employment. It also unintendedly impacted on the implementation outcome of feasibility.

The efforts to integrate IPS into the mental health services was a strategy and a planned adaptation, aimed to support feasibility. These efforts by the steering group and ESs were possible mediators consistent with core functions. However, ESs adapted to team meetings where service users were absent, limiting inter-professional collaboration. While this improved the implementation outcome of IPS acceptability at the mental health service, it also undermined the core functions of integration as well as following the service user´s preferences. Fidelity ratings for team integration were high, as the ESs attended team meetings and encouraged staff to actualise service user´s work planning. However, analyses revealed a mismatch with actual practice. Fidelity ratings were ascertained to be too high in relation to how the workflow developed. Moreover, ESs’ time spent adapting to internal workflows reduced their presence in the community, as a ripple effect, limiting the core function of employer engagement and unintendedly affected the intervention outcome competitive employment.

## Discussion

Making adaptations to the context when implementing interventions in practice have been reported as being necessary in most cases [1, 2]. The findings in this study underline that some adaptations could enhance, or mediate, and align with implementation outcomes, while others obstruct core functions, intervention and implementation outcomes even though the purpose to adapt was sympathetic. The context in which an implementation takes place influences and interacts with both the intervention and the implementation process continuously and in multiple ways [33]. IPS was adapted to the contexts of various stakeholders, which can be understood in light of the fact that the model is implemented within an implementation structure [34], where several different organisations must be involved for employment support to function effectively. In such context, responsibility for vocational rehabilitation is distributed across multiple organisations, and effective collaboration within this network is required to ensure optimal support for individuals seeking employment. Adaptations to these external organizations in the Swedish and international IPS contexts are well known [35], and highlight the complexity of interventions requiring multi-stakeholder collaboration [20, 36–38]. This contextual landscape constitutes part of the complexity inherent in implementing an intervention in which different actors operate under distinct mandates and regulatory frameworks [11, 35, 39]. Applying the MADI framework retrospectively made it possible to analyse these adaptations, and it enabled an identification of adaptation pathways and how far these adaptations were made, influencing both intervention and implementation outcomes. This aligns with previous research, which has similarly demonstrated that the degree of adaptation can affect core components [40]. Adaptations occasionally exceeded the prescribed instructions of an intervention, thereby influencing its core functions; however, these adaptations might consistently be made with the intention of benefiting service users in different ways. This finding mirrors the behaviours and intentions demonstrated by ESs, who sought to facilitate the IPS process for clients.

The findings highlight the importance of assessing whether core IPS functions are compromised, as this risks weakening the evidence base. While ecologically valid adaptations aim to align with organizational contexts and support service users, they drifted too far in some cases—disrupting key elements like rapid job search and employment outcomes. As Pérez et al. [23] emphasise, identifying minimum fidelity requirements is crucial. Domain 2 in the MADI framework supported both identification of how different actors in an implementation could act as a mediator or moderator and to understand why these adaptations were made [2]. To maintain its integrity, IPS must avoid over-adapting to external systems while preserving its flexible, user-centred approach, and as previous research suggest, fidelity and adaptations need to co-exist [1]. In this study IPS fidelity scores often aligned with IPS principles, but some diverged from the core functions. High fidelity does not always reflect true adherence, highlighting the need for deeper analysis of these complex relationships. Discrepancies may stem from limitations in fidelity assessments or their inability to fully capture contextual adaptations and why they are made. As discussed by von Thiele et al., and other authors [1, 23], it is essential to evaluate adaptations in relation to both outcomes and the intervention’s theoretical foundation. Fidelity tools must be tested across diverse contexts, and in some cases, the context itself may need to adapt to the intervention [1]. In Sweden, the entrenched SPES work support model often forces IPS to adapt around individual caseworkers or find creative solutions, which undermines equitable access to work rehabilitation [12]. Understanding which adaptations support or hinder implementation and intervention outcomes is critical for guiding future implementation. The combination of the three domains in the MADI framework helped the analysis to clarify the intention behind the adaptation and how it impacted [2]. The analysis showed that the ESs had strong intentions to adhere to the core functions: to help participants enter employment quickly, to ensure that everyone who wished to take part in IPS was eligible, and to follow the participants’ preferences. In the program fidelity assessments, this work was therefore perhaps rated highly, but at the same time, the analysis showed that adaptations were made to the context that unintendedly affected intervention outcomes related to core functions. These influences were not captured in the fidelity assessments themselves, even though they were discussed in the steering group and among the employment specialists.

From a theory of change perspective, implementation planning requires shared goals and a thorough analysis of each organisation’s needs, including necessary modifications to rules and routines related to return-to-work processes. Each organisation must identify the specific change outcomes required to deliver work support according to IPS [39]. Because the SPES is involved in multiple procedures for jobseekers, it must be able to accommodate IPS principles and undertake structural changes aimed at minimising adaptations toward stepwise approaches. This involves analysing each stage of vocational rehabilitation to identify components in need of modification. Applied prospectively, the MADI framework could support such analysis before implementation begins [2]. However, no such systematic planning took place in this project. Despite steering-group discussions, extensive vocational rehabilitation experience and the SSIA representative working intensely to make changes to benefit the service users, there was no shared understanding of the organisational changes required, and SPES representatives lacked the mandate to initiate them. Some necessary changes would also have concerned legally regulated procedures requiring system-level decisions [33], which were beyond the scope of this project.

Differences in organisational norms and value bases also shape implementation efforts. IPS is grounded in values of social inclusion, individual strengths, and personal recovery—including through participation in employment [41]. These contrast with the stepwise vocational rehabilitation model, which is rooted in a medical logic emphasising assessment and treatment before employment based on evaluated work capacity [12, 42]. Such divergent value bases, documented in IPS implementation research [42, 43], must coexist and be meaningfully integrated [44]. This requires open discussion between organisations and treatment staff during the planning phase, including careful consideration of the implications of such a transformation [39].

The personal recovery perspective was not sufficiently addressed during training in the mental health services in this project and there were also signs of resistance to change from some of the professionals. Such resistance may stem from professional mindsets, limited resources, and organizational norms and traditions [45]. Traditional roles in the mental health services often focused on medical treatment which conflicted with the recovery-oriented approach that IPS promotes [12, 16]. Resistance to change during the implementation process in mental health services could arise at the individual level, as some staff did not fully accept the introduction of IPS, making adoption difficult. This created challenges for ESs in collaborating with staff at some of the services, as required by IPS principles. Such resistance may stem from negative emotions toward anticipated change, doubts about whether the model aligns with perceived professional responsibilities, or concerns about one’s ability to manage or participate in the change [46, 47]. It may also result from a failure to recognise the benefits of the intervention.

This underscores the importance of identifying staff attitudes and willingness to engage, as resistance can affect both client outcomes and the implementation process [13]. Earlier studies indicate that shifting attitudes, establishing shared values and goals, and developing collaboration between mental health staff and ES require time and deliberate effort [16]. Implementation research underscores these challenges, noting that even with well-established determinants of successful implementation [48, 49], leaders and staff must recognize that introducing a new intervention and collaborating across organisations inevitably alters workflows, organisational routines and even values.

### Limitations

This study used an embedded case design to explore contextual adaptations during IPS implementation in five mental health services, capturing both organizational and individual perspectives. The project was followed over two years using a case study protocol to ensure trustworthiness [50]. Due to the Covid-19 pandemic, some interviews in 2019–2020 were conducted via Zoom, which occasionally affected audio quality and transcription accuracy. These recordings were reviewed multiple times to minimize errors. All other interviews, including those in 2021, were conducted in person, and steering group meetings were attended that year.

A total of 26 informants from various professional roles and organizations were interviewed, supporting the study’s credibility. However, the absence of interviews with representatives from the employment and social insurance authorities is a limitation, though their actions were observed in steering group meetings and documented in memos and notes as well as in steering group protocols. The employment service’s limited participation, due to internal restructuring, affected both the project and the research as their voices and experiences could not be captured in this study, but their actions and the difficulties they faced in being engaged in the project could be noted through the work of the steering group.

Multiple data sources were collected and analysed chronologically to identify patterns and decision-making processes. Applying the MADI framework through deductive analysis helped clarify the nature, context, and effects of different adaptations, enhancing understanding and potential transferability [26]. While the embedded case study approach grounded the findings, results from five local mental health units may not be generalizable to other settings.

The MADI framework has also been used in pilot studies, one of which [51] shows that during a pilot phase it is possible to use the framework to identify which adaptations may be necessary to better align with the intervention, as well as to detect adaptations that could potentially influence outcomes and thereby mitigate such effects from the outset. In future studies on the implementation of IPS, the MADI framework could be used in a similar manner, serving as a foundation during the planning phase to guide work on change strategies and change-oriented perspectives.

## Conclusions

This study shows that using the MADI framework to analyse both planned and unplanned adaptations during IPS implementation offers valuable insights into how these changes affect the intervention. It could help stakeholders understand adaptation pathways and intervene when needed to preserve core functions and effectiveness.

Adapting IPS to local contexts is often necessary, but excessive changes can compromise its core functions and values and reduce its impact—evidenced by fewer participants gaining employment compared to previous IPS studies in Sweden and Europe. High fidelity scores may not reflect true adherence if adaptations alter how core functions are delivered. MADI could usefully be applied in more studies on IPS, not only to facilitate implementation but also to enable comparison across contexts and countries. This could provide a solid systematic knowledge base for understanding how implementation unfolds internationally.

## Supplementary Information

Additional file 1.

Additional file 2.

## Data Availability

The data sets used and analysed during this study are available from the corresponding author on reasonable request.

## Abbreviations

IPS: Individual Placement and Support
MADI: Model for Adaptation Design and Impact
FINSAM: Financial Coordination Association
ES: Employment specialist
SPES: Swedish Public Employment Service
SSIA: Swedish Social Insurance Agency
SEFS: Supported Employment Fidelity Scale
