# Supplementary material for: Contextual adaptation, implementation, and outcomes of individual placement and support: a case study
Source: Implement Sci Commun. 2026 Feb 5;7:40. doi: 10.1186/s43058-026-00875-5 (PMC12930962; doi:10.1186/s43058-026-00875-5)
Supplement: Supplementary file 1 — Additional file 1. [file 43058_2026_875_MOESM1_ESM.docx]

**Supplementary material: Selected questions from the interview guide**

Tell me about IPS and in what way it was introduced here.
Describe the background of the project, describe the process of preparations and planning.
Describe your service and the interventions that you can provide – what other work‑oriented interventions do you have?
How do you view IPS as a method compared to other interventions?
Do you/you all have previous experiences of starting to work with a new method?
What conditions do you have for running the method? Are there challenges or facilitating factors that make the work easier within the organization? Please describe.
What does collaboration with other actors look like?
How has the cooperation around the project developed?
Does IPS need to be adapted in any way in order to gain a foothold with collaboration partners? Please describe

How do you view stepwise improvements for the client compared with IPS?
Do you have confidence that stepwise interventions support the client? In what way?
Is IPS established within your administration now? Are there other administrations that claim the method?

How do you motivate the staff to work with the method? Examples.
Do you associate any values with IPS? What type of values do you associate with IPS? How do you think these values are expressed through the practical work with IPS?
What has the introduction looked like for you at the clinic?
Tell me if you see any challenges in working as an employment specialist within psychiatry.
Tell me how the integration into psychiatry has gone during this project. Have you adapted to the clinic? In what way, if so?
Do you/you all have previous experiences of starting to work with a new method?
How do you view IPS as a work‑rehabilitation method?
Does IPS, as you know the method, correspond to your professional role and job description? In what way/not?
Have you/you all needed to adapt the method to different organizations in the work with IPS?
Have you encountered any resistance from staff in other organizations? Tell me.
