# Supplementary material for: Contextual adaptation, implementation, and outcomes of individual placement and support: a case study
Source: Implement Sci Commun. 2026 Feb 5;7:40. doi: 10.1186/s43058-026-00875-5 (PMC12930962; doi:10.1186/s43058-026-00875-5)
Supplement: Supplementary file 2 — Additional file 2. [file 43058_2026_875_MOESM2_ESM.docx]

**Supplementary table, quotes from interviews**

**Table 3:** Quotes from informants including employment specialists, treatment staff in mental health services, project leader and managers. Ten employment specialists attended two different group interviews. In the quotes beneath, six of the ES are represented and selected. The quotes refer to the three domains in Model for Adaptation Design and Impact (MADI); adaptation characteristics, possible mediating and moderating factors and implementation and intervention outcome.

| **Informant** | **Quote** |
| --- | --- |
| **Adaptation to SPES rules and resources** |  |
| Employment specialist | “But then I think… then there is this thing with long internship periods, now since it is so complicated with the Employment Service as well, I mean like if they are going to be coded and you’re supposed to see what adaptations they need and so on, then you do need an internship period to sort of find the right fit there. Mm. Like what… what support the participants need and so on. And that they get to feel that, but then… I think that… that it is important not to have too long internships either, that…” |
| Employment specialist | “Yes, there have been very many twists and turns now, so I might sum it up a bit. But then it dragged on and we had a meeting that was booked by the Employment Service, when he and I met and we sat and waited, no one called. Then he got a letter at home saying that he had not been reachable and that it could, like, lead to financial consequences. Now that was wrong, so the Employment Service backed down there and he got a new appointment, and then we decided again that yes, well, then we’ll meet so that we are both part of this phone call. Then they call an hour earlier, so he had to take that call himself and didn’t really know, like, okay, what was this now then, that they had said that in three weeks I will get an in‑depth meeting. And we had an employer then, where we had planned that he would start an internship and we had written it for three months, as we have done earlier with other people too, and it hasn’t been any problem, and it was, like, very clear that there were good chances for employment afterwards. But then there at the beginning of the summer the money at the Employment Service apparently ran out, so then it wasn’t possible to approve the internship.” |
| Employment specialist | “And I can say it like this, this is kind of an eternal topic of discussion that we keep dealing with. Yes, I get tired when it comes up again. Mm. Yes, but it’s… the cooperation with the Employment Service is really tough and really difficult. I mean, if someone has activity support or unemployment benefits and you have to sort of go into all that, it becomes really hard.” |
| Treatment staff | “But the way it works for me, for example, if I start from that. I mean, the only obstacle is, again, this thing with different authorities.  Because it is… the Public Employment Service are the ones who hold all these state subsidies, and then maybe they have a different idea of how it should be done.  Or that it becomes some kind of clash with the municipality, that the municipality says no, but we have these requirements if people, for example, have social assistance. That it can… sometimes it gets a bit strange and rigid in the system, that you get stuck… I mean, it somehow gets stuck because of that.  Yes, but that has absolutely happened, that some feel frustration and a bit of low mood because it’s not really moving forward, and I had someone who… who ended up really very much in-between and was basically forced to meet another employment specialist, because that was the requirement in order to get a paid internship at a place. And then it gets delayed and the Public Employment Service cannot make a decision, and they have to wait until you’ve been registered here and there and there and… so…” |
| Employment specialist | “Yes, it’s like that… you have to work in parallel here, like, with the contact with the Employment Service and then the employer, and you also have to keep the employer in a good mood and the participant in a good mood if things get tangled at the Employment Service. The processes… it’s like… it’s hard to achieve, you know, jobs quickly when that part gets tangled. Then you end up… it drags out longer in time, to getting a job.” |
| Employment specialist | “I mean, it is precisely these delays, I would say, that are the absolutely biggest problem, because I still experience that when you work with IPS… and in the role that we work in, generally speaking, our job is somehow to be like, okay, what are the frameworks, what are the rules that exist, and then we find a way to work around them like that. And we have always done that, but now it can take a month before I find out what framework I have to relate to. And then you become quite paralyzed. Because the last thing you want to do is also to do something that in the long run would be bad for a participant, you know.” |
| Financial Coordination Association | “And then I think that we have helped with small questions as well, how the Public Employment Service makes its decisions, we are kind of spokespersons for our projects, towards our members, so that when things don’t work with the Public Employment Service, when things don’t work with the social services, we use our meeting formats and channels to raise these issues.  However, it gets a bit difficult when, for the fifth time, we ask the Public Employment Service a question about something, when they maybe haven’t really delivered or it’s difficult to get in touch with them or so on, so it becomes a bit embarrassing for them, too.  So we… we try to use the channels that we have, to influence things, simply. But it’s not always possible, considering how the organization looks among our members at the Public Employment Service, for example.” |
| Financial Coordination Association | “Well… I think that in general this kind of… yes, government agencies, they have their mandate, national mandates from the government very clearly, and this thing about working locally is not their strong side… and when it was… they used to have local county branches and we had a much clearer local arrangement, and then it was easier, but the problem then was that it looked so different across the country, so that was why one… it became national. It is also partly an unreasonable situation for an employment officer to manage to focus on a small project that becomes an exception.  The way our operations must still be viewed in the larger picture. The Public Employment Service’s focus has also been on those target groups who have unemployment insurance, and ours usually do not, so there are somewhat different priorities.”  “No, we don’t have time for that, we have these large target groups that we are supposed to work with…they cannot… they cannot allocate an employment officer like that and place them in a smaller operation with a certain target group.” |
| Project leader | “Yes, but it… it is uneven, I think. Yes, but the Public Employment Service has just become like a source of misery, almost exclusively, it is… I mean, it is very sad to say, it is still an agency that is needed, it is one of those important agencies that an IPS operation depends on being able to collaborate with. And the last… the last two years this has not been working, since the collaboration with the Public Employment Service has actually become increasingly difficult. …but as time has gone on, we have… it has only deepened, how big the gaps in the collaboration have become.” |
| **Adaptation to stepwise social insurance structure** |  |
| Employment specialist | “But those who do not… if there is someone who does not have contact with the Social Insurance Agency, then there can be major challenges when it comes to collaboration with the Public Employment Service, I think. … So that is how you feel that yes, if the person has sickness benefit or, like, compensation from the Social Insurance Agency, yes, what a relief, or income support, then it also becomes much easier.” |
| Project leader | “But that in turn, unfortunately, I believe has to do with… the treatment staff, I think, get very much drawn into functional assessments because the caseworkers in social psychiatry demand it, for the person to be entitled to various interventions. So it is about… you have to see this in some kind of system as well. We are in a big city and scarce… well, resources, yes. And that we have built ourselves into very extensive assessment systems, and then it becomes like this. And that… that is really bad.” |
| Project leader | “So then it felt… then it felt really good. And then we have had a real workhorse from the Social Insurance Agency in our steering group, who has truly brought the questions with him when problems have arisen. So there… he has done what he has been able to, really, and maybe almost a bit more, as he has even gone and influenced and sort of pushed the regulations almost, so that we can do what we need to do in the project, so that has been really fantastic. Sometimes you need to see it with a bit of a time perspective as well, in order to understand how much he has done.” |
| Treatment staff | No, but exactly, but of course it… it is really sad like that for the patients many times, because they want to move forward, if they have been at the information meeting and then they want, like, to move on, but at the same time it is also part of the process, it can be a bit good that yes, well now there is this queue and that is how it is everywhere in all these different… I mean the Social Insurance Agency also has queues for its collaboration with the Public Employment Service, so there are queues everywhere.” |
| **Adaptations to mental health service day-to-day workflow and case management** |  |
| Financial Coordination Association | “It says, at least in most of our projects, to work with the implementation, but it’s not really being done… so I think that the project leader really has a… really has tried with an implementation group, but above all what I have assisted with is helping a bit with how things look in the City County with the Health and Medical Care Administration and with the City County Healthcare Area, that is, what levels exist and which channels one can turn to in order to get… and it really is a bit of a jungle, how the city is governed, so that’s one example.” |
| Project leader | “And this is the thing, this is what you see, that it’s about… the ES is the same employment specialist with all these people, so it’s this factor, that this engagement from the team is needed.  Without that then… then it’s not going to fix everything, I mean it’s not like… it’s not about lack of nuance here, these are still nuances, but if something is going to happen with those people who don’t have their own drive, then there has to be an engagement from the healthcare staff, I mean that… that I am prepared to just, like, say.  So we decided today, since we are going to have a local project group meeting there, to really bring this up on the table for the local project group, like what is actually the integration of IPS at the clinic, to really talk like this now that we have done this fidelity measurement. … I mean, one might think this is a good thing, but one might still not really have grounded in what is my part in all of it.” |
| Employment specialist | “I did not know anything about the mental health service, I have never been working in those services. It is a completely different way to work.  With time clocks and point‑chasing and that whole thing they have, and they have their care processes, and they are pretty much set and so on, so I don’t know if… I mean it’s not so much about adapting IPS, I don’t think, because there you have a method that we are supposed to work according to, but I didn’t feel that I knew “this is how I should do” when I came out, instead it has had to be adapted to how the clinic has looked and their routines and what the team looks like there and so on.” |
| Employment specialist | “Yes, I got… when I started I had… we had a list at the clinic and above all because one service then joined when the project was already running, it was one of the clinics. So there was a list with fifteen people, not all of them ended up being eligible, and the system still works mostly like that today as well, that you put your name on that list and then you keep track of when then. But back then it was more or less an info meeting, and if the person said yes, then you started.  And today I try to determine much, much more whether the person is ready, what the situation looks like. Then it is also… it also ties into a lot of what has happened with the Public Employment Service and everything else, that today it is very much about figuring out what interventions the person has, whether we even have a workspace here or if we have to follow some planning that already exists somewhere else, to see if it is relevant, so to speak. So today I can have two–three–four info meetings with the same person, together with the case manager.” |
| Employment specialist | “Yes, but certain people, not staff groups as such, because like half of the group of case managers were positive and interested, and then the other half were like, well, this doesn’t work, or it hasn’t worked, you know. So there was no general picture, but I think that some simply found it harder to take in what this is, many have worked at this clinic for, like, forty years and, you know… and then IPS is very new, if you think about how long they have been there and seen things come and go, I mean even… even the occupational therapist and the physiotherapist have also had difficulties, you know, getting into it, but now… it is more well‑known now what they are needed for, but they have also had to fight their way in a bit, like, we are here, we can make assessments and…” |
| Manager | “And then of course we have individual contacts with them, it is the case manager who coordinates things, who is the spider in the web with our patients. We have roughly five hundred patients here in our unit. And then there are seven case managers who… who take care of them, so they each have somewhere… yes, whatever it might be… eighty patients, or seventy–eighty patients per case manager.  For each patient, there is of course a physician responsible for the patient and a case manager as the basic setup, so to speak, who they… and then it is at the treatment conference that you to a large extent involve the other professions. When you feel that yes, but here is someone where we need support and help from the psychologist and to do an assessment about whether there is something else that we don’t quite recognize, then you need to do an assessment regarding this individual.  It is also a bit specific with our patients, with the problems they have with psychotic disorders, so of course it is not entirely easy to get them into a new project, so to speak.” |
| Treatment staff 1 | “I mean, this thing… it kind of clashes with this “I want to start working now,” “yes, we can help you in eight months.”  Yes, and how do you think it’s going to get participants from your clinic into the project?  I mean, in the project it hasn’t been difficult at all, there are many who have been interested. Then I know that the endurance is a bit so-so, but that probably depends very much on the current circumstances. But it is difficult to squeeze people in at the moment. So our IPS coach or employment specialist, or whatever it’s called, really has to work hard. But I think that we bring it up more often when we meet, because I mean there are many who are… everyone has a contact person, except with the doctors who have the overall responsibility, but… but that all of us who are contact persons, that we maybe bring it up a bit more often, like “how is it, do you… do you miss starting to work or would you like help with that.”  “I don’t think that has been done as automatically before, instead you might have talked a bit more like “well, do you have boring days, are you isolated, well, there is occupational activity, we can call the municipality.”  That you have automatically thought that that is where they should turn. And they (another service; author comment) meet much more often as a team together with the patient than we do, for example, and then I know that we have another team where they are very much like small individual islands and the only time you have an overview of patients and share things is when you meet the doctor, which you do… often, but… but that… so we probably structure it very differently and we mix… we mix with our patients in different ways, simply and the patient is not present at the team discussions.” |
| Employment specialist | “Yes, but I have also discovered during a period, because it is a bit different at the clinics. It was like this that at my clinic they have had limited teams in order to have fewer people in the room. And what happened then was a bit interesting, because then it became… the psychologist wasn’t there, the occupational therapist wasn’t there and I wasn’t there, so then you were back to the core group. And there is a bit of a pull towards that, that it is medication, it is treatment, it is… and the risk with that is that it becomes this ordering system.” ‘Yes, we think this person would be suitable for work. Can you take (ES)… here now.’  And… but you can also turn it around, because now I am also in the situation where it’s starting to get full, like it… and then I have raised it, that I want us to bring it to the teams, not that we sort individuals out or assess work ability, but that there must be some order in this, because then some feel that they have talked to me… *laughs*… and booked a… like it… so it’s about finding something in this, and that it should belong to the team… I want them to discuss it, and sometimes they have so little time to discuss.” |
| Project leader | Yes, but it’s… yes, but it’s still this thing about talking about… and I don’t know if it’s my role, I mean this is also what drives me a bit crazy, is it my role to talk psychiatric rehabilitation with these clinics, I don’t know. Sometimes I can feel a bit, like, presumptuous coming in and saying what they should do. Mm.  But that is still what we are sort of tiptoeing into now, it’s unavoidable right now, because that’s where we are. Because we can see that around some IPS participants there is great resource‑group work around the person, and around some others not.  Mm.  And that doesn’t depend on the employment specialist, but on the rest of the network around them, and then you have to… in some way you have to be able to address that issue, but I think this is… it’s… it’s not like it feels very easy, that I’ve decided to start with that next week at one of the services.’’  I mean, we have talked about this, but we have still talked about it a bit more, like, disguised as team collaboration and that good collaboration around the individual benefits IPS.  But if you sort of peel that away and start talking about what is needed, because that is where we are going now, now we peel off one more layer, we remove those, like, words and start looking more at what it means.” |
| Employment specialist | “So I understand in a way that they are so set in that yes, when a case manager or someone goes in one morning, then they immediately see like some patient has called in sick or they are admitted, or something has happened during the weekend, and they don’t need to talk to everyone, or the doctor, about what has happened, because they just see everything in a second, whereas that really gets lost, I mean I think I have been in some situations like that where I feel that yes, maybe I was forgotten there, I mean I’m not forgotten very much when it’s… when things are running smoothly. But when it doesn’t go well, then somehow… then they go into their own thing.” |
| Treatment staff 2 | Yes, exactly, because I was working there when an employment specialist arrived, and then I had already kind of made a list, these ones I think, based on the knowledge I had then about IPS, it was like yes, well these ones I think are probably closer to work, so that they can take a job quite quickly and want to. So I kind of thought that yes, well these ones… I’ll hand over to IPS, that it would fit well, whereas these others who maybe need an activity first, or yes, that it’s like… some… I mean it can be that you just inform them that these opportunities exist and if you are on sick leave you can go through the Social Insurance Agency, there is something called coordinated work‑oriented rehabilitation in collaboration then with the Public Employment Service and the Social Insurance Agency, and then it can take another month before they kind of start approaching that.” |
| Employment specialist | “Yes, but I mean I can notice a difference with the people who… we have two floors where I sit, and luckily all the case managers sit on the lower floor, and now when I just think about it, I have much less spontaneous contact with those staff who sit on floor two. Because I’m not up there running around in the same way. I would have been in and talked much more with them if they sat on floor one. So this closeness is like a physical closeness, at least for my part, or geogra… yes, being on site and nearby.” |
| Treatment staff 2 | “I mean, I do think that there is quite a lot of treatment focus overall, I do. A lot is about medications and so on, and of course those are important parts, but then to be able to bring this in, I mean to be able to guide the patients out into society, I think that maybe that… that there isn’t… you don’t have… there is too little focus on that. I mean sometimes you talk about it, but many times the focus is on this, because I mean it is… you don’t have that much time and there can be medication adjustments which are also of course important and so on, but just this thing with how, what does this person do during the day, I mean how can you introduce something, is there any group at the clinic they can join, or how should you think, should you contact the Social Insurance Agency, is there any activity available and so on. That many times it is perhaps the patient who has to ask about it for someone to, like, pick up the ball, instead of having… being able to offer that this exists.” |
| Treatment staff | “Yes, but I mean that we… because we talked about that, that yes, we would sit down, that we go through which people I might work with and which are suitable for IPS, that we should do it together a bit. Now it was like, since I was at two clinics as well, so of course I couldn’t be involved all the time, and there were like three IPS… employment specialists then, because there were two down at the clinic for newly emerging psychotic illness and then one up there, so… We didn’t quite manage either, but they had their waiting list and so on, and I had my way of working, so I mean more around that, like how are we going to… how do we do this in the best way, how should we think and who… because we had… we all had challenges around how, I mean, how can you approach and reach the Public Employment Service, the Social Insurance Agency and so on.” |
| Employment specialist | “That it goes easily, you know, you can just knock on the door or talk to someone like that. I also work half‑time in the regular municipal service, and it becomes… when you don’t have any kind of established collaboration with a clinic, it always becomes so much hassle, you have to call and arrange something and it takes a long time, but here it goes very quickly.” |
| Employment specialist | “In the same way as those out at the clinics, they get that from us sitting there with them, but I’m thinking specifically about things like medications and so on, well, I’ve been in a number of situations where maybe suddenly someone doesn’t show up for their internship one day, or something, and I bring it up in the team really quickly, because I’ve found out about it really quickly. Yes, well, then it turns out the person has changed medication. If I had been sitting… now, I’ve never worked in the regular municipal service here, but when you’ve worked in similar services in the municipality, it can take several weeks before you find out about such a simple thing, and now it was like this, yes, well, then we don’t need to look for issues with motivation or anything, yes, these people are on strong medication and it becomes very clear.” |
| Employment specialist | “Yes, and I think that you… or at least I experience it like that, that you force them into a much more non‑prestigious way of looking at things, at least… I mean the clinics are very… doctors have quite high authority, then you have case managers, at least that is how it is at our clinic, who own the case in many ways, it is their case, but when we are involved… I mean it doesn’t really work like that when we work, you cannot have someone who owns the case, but it has to be this more team‑based way.” |
| Case manager | “But I would like to say that we haven’t really… and then of course Corona has also played a part, that we haven’t been able to have any meetings or anything, so we… right now we are in the process of finding a… some kind of work routine for how we should allocate the patients to our… to our job coach.  But… we… we have our, like, group of patients and then you partly, of course, based on the patient, what they want, apply for that intervention, so to speak. And then we usually… it is quite popular, so there are many who want it, and I know that ES has a small list that she is working through, but there we haven’t really, like, come… found a good routine for how we should build up this list, the waiting list, so to speak.” |
| Project leader | “I got another thing to think about when I saw a compilation of statistics from this quality register, because then you can still see that one clinic stands out in this context, it is the clinic where they have the fewest people, at least reported statistics, where they… it is the clinic where they have the most people who have no kind of activity or occupation at all.  So I think that does say something about… the foundation… the basic situation at that clinic, that there are many there who fall outside, who perhaps do not have what one needs.  And if I’m going to be very frank, I think that… they maybe don’t have any really clear idea of what psychiatric rehabilitation is, at that clinic, I think that is what’s missing.  They have some kind of idea about what treatment responsibility is, which the case manager is responsible for, but I don’t think that… I don’t hear that they think in terms of psychiatric rehabilitation.” |
| Project leader | “At the same time, not everything is in place, and I’m thinking that it is precisely this close, somewhat seamless collaboration. Where you take on different roles, but you have a shared goal that we do what we can to support this person in that person’s plan moving forward. That is when it becomes really good, because that is when things can happen, because you can also see that when the employment specialist becomes quite alone, you might have a small formal collaboration at the clinic, but in terms of content there may not be much drive in it, then it is much more difficult.” |
| Project leader | “Yes. So it… it is this kind of cooperation. I still think the level of ambition from the region’s side, from these five clinics’ side, has been that they want this and they have made an effort, and we have had structures that have meant that we have… we have had different channels to talk about how the cooperation works and what the next step is, what we need to do. Then it has looked a bit different at the different clinics, but there we have at least had structures, and we have been able to make ourselves heard.” |
